# Associations between Disease Awareness and Health-Related Quality of Life in a Multi-Ethnic Asian Population

**DOI:** 10.1371/journal.pone.0113802

**Published:** 2014-11-26

**Authors:** Kavita Venkataraman, ChinMeng Khoo, Hwee Lin Wee, Chuen Seng Tan, Stefan Ma, Derrick Heng, Jeannette Lee, E. Shyong Tai, Julian Thumboo

**Affiliations:** 1 Saw Swee Hock School of Public Health, National University of Singapore and National University Health System, Singapore, Singapore; 2 Department of Medicine, Yong Loo Lin School of Medicine, National University of Singapore and National University Health System, Singapore, Singapore; 3 Department of Pharmacy, National University of Singapore, Singapore, Singapore; 4 Department of Rheumatology & Immunology, Singapore General Hospital, Singapore, Singapore; 5 Epidemiology and Disease Control Division, Ministry of Health, Singapore, Singapore; Mario Negri Institute for Pharmacological Research and Azienda Ospedaliera Ospedali Riuniti di Bergamo, Italy

## Abstract

**Background:**

Health related quality of life (HRQoL) is an important dimension of individuals' well-being, and especially in chronic diseases like diabetes and hypertension. The objective of this study was to evaluate the contributions of disease process, comorbidities, medication or awareness of the disease to HRQoL in diabetes mellitus, hypertension and dyslipidemia.

**Methods:**

This was a cross-sectional study of 3514 respondents from the general community in Singapore, assessed for HRQoL, disease and comorbid conditions through self-report, clinical and laboratory investigations. HRQoL was assessed using SF-36 health survey version 2. For each condition, participants were categorized as having 1) no disease, 2) undiagnosed, 3) diagnosed, not taking medication, and 4) diagnosed, taking medication. Analysis used one-way ANOVA and multiple linear regression.

**Results:**

Diagnosed disease was associated with lower physical health component summary (PCS) scores across all three conditions. After adjustment for comorbidities, this association remained significant only for those not on medication in diabetes (−2.7±1.2 points, p = 0.03) and dyslipidemia (−1.3±0.4 points, p = 0.003). Diagnosed hypertension (no medication −2.6±0.9 points, p = 0.002; medication −1.4±0.5 points, p = 0.004) and dyslipidemia (no medication −0.9±0.4 points, p = 0.03; medication −1.9±0.5 points, p<0.001) were associated with lower mental health component summary (MCS) scores. Undiagnosed disease was associated with higher MCS in diabetes (2.4±1.0 points, p = 0.01) and dyslipidemia (0.8±0.4 points, p = 0.045), and PCS in hypertension (1.2±0.4 points, p = 0.004).

**Conclusions:**

Disease awareness was associated with lower HRQoL across the diseases studied, with PCS associations partially mediated by comorbidities. Equally importantly, undiagnosed disease was not associated with HRQoL deficits, which may partly explain why these individuals do not seek medical care.

## Introduction

Health related quality of life (HRQoL) is an important dimension of individuals' well-being. The measurement of HRQoL is particularly relevant in the management of chronic diseases which are known to be associated with impaired HRQoL over extended periods [1], [2] and where the treatment outcomes should include perceptive benefits to patient mental and physical health as well. Diabetes mellitus and hypertension, the two most prevalent disorders worldwide, have been reported to be associated with modest reductions in HRQoL [3]–[8]. However, most of the studies linking HRQoL to these diseases have been conducted in individuals with previously diagnosed disease. Also most studies have not evaluated associated comorbidities completely. Therefore, it is not clear whether the reported reductions in the HRQoL in patients with already diagnosed diabetes mellitus and hypertension are due to the disease process itself, comorbid conditions, therapeutic interventions and/or awareness of the disease.

The impact of awareness may be particularly pertinent given the widespread implementation of screening programs for diabetes mellitus, hypertension and dyslipidemia. Several studies assessing the impact of awareness of disease on HRQoL in newly screen-diagnosed individuals have yielded inconsistent results. Studies involving screening for diabetes mellitus have found that when diabetes is diagnosed through screening, there is a short term increase in anxiety but not in HRQoL measured in the longer term [9]–[11]. The diagnosis of hyperlipidemia was also found to have no negative effect on mental health 25 years after diagnosis [12]. On the contrary, compared with undiagnosed individuals, those with diagnosed hypertension have been found to have poorer HRQoL [13], [14].

We recently examined the association between diabetes and hypertension and HRQoL in a Thai population [15]. The presence of diabetes mellitus and hypertension was associated with lower physical component summary scores (PCS). However, there was no association of PCS with awareness, or treatment for these conditions. In contrast, mental component summary scores (MCS) were lower in those who were aware of the diagnosis of diabetes mellitus or hypertension than in those who were unaware of their disease status. However, this was partly because those who had diabetes mellitus or hypertension but were unaware of it had higher MCS than those without disease while those who were aware of the disease had slightly lower MCS. This finding, of higher MCS in those with undiagnosed disease, has not been replicated in other populations. Furthermore, our previous study did not comprehensively assess all the complications associated with these disorders to allow adjustment for these complications. This is particularly important given that we have previously reported that the reduced HRQoL associated with diabetes mellitus is primarily related to the presence of diabetes related complications [16]. As such, the presence of comorbid conditions, by making the diagnosis or treatment of these diseases more likely, may confound the association between awareness or treatment for these disorders and HRQoL. In addition, treatment for hypertension appears to negatively affect HRQoL among those diagnosed with hypertension [17].

We therefore aimed to determine the contributions of disease awareness, treatment and comorbid conditions on HRQoL in chronic disease by using data from a health survey in which individuals were classified as having disease based on history (previously diagnosed) and objective indicators (undiagnosed) along with detailed assessment of comorbid conditions. We focussed on three common chronic medical conditions, namely diabetes, hypertension and dyslipidemia.

## Methods

### Study design and participants

In this cross sectional, Institutional Review Board approved study, a total of 10,747 individuals who attended one of four previous cross-sectional surveys (the Thyroid and Heart Study (1982–1984) [18], the National Health Survey (1992) [19], the National University of Singapore Heart Study (1993–1995) [20] and the National Health Survey (1998) [21], were invited for a follow up survey between 2004 and 2007. We used data from the follow up survey for this study.

Details of participant recruitment and data collection have been previously published [22], [23]. Briefly, participants from a list provided by the Ministry of Home Affairs were contacted for home interviews by trained field interviewers. Of the 7774 subjects who were contactable, 30 refused consent, and 7744 completed the health questionnaire. All interviewed participants were invited to attend detailed clinical examinations and collection of biological specimens subsequently. These examinations were conducted by trained research nurses and measurement technicians using a predetermined and pretested protocol, shortly after the home visit.

### Ethics statement

Ethics approval was obtained from two institutional review boards (the National University of Singapore and the Singapore General Hospital) before study commencement. Informed written consent was obtained from all participants before conducting the study.

### Data collection

Socio-demographic characteristics were captured using interviewer-administered questionnaires. Ethnicity was recorded as Chinese, Malay or Asian Indian; marital status as never married, currently married or separated/divorced/widowed. Education level was categorized based on years of schooling as <7 years, 7–10 years and >10 years of schooling. Working status was classified as working or not working. Smoking was categorized as current smoker or current non-smoker and alcohol intake as current drinker and current non-drinker.

For the health examination, participants were examined in the morning after a 10-hour overnight fast. We collected anthropometric data and clinical data which included blood pressure, blood glucose, lipids and serum creatinine, and urine albumin creatinine ratio. Peripheral neuropathy was detected by foot examination using monofilament and neurothesiometer. Ankle brachial index was used to identify peripheral arterial disease and fundal photography was carried out and graded for the presence of retinopathy During the period 2 April 2005 to 20 February 2006, neurological foot examination, measurement of ankle brachial index and retinal photography were conducted only for every alternate Chinese participant, but for all non-Chinese participants, because of limitation of resources. Details of health examination, blood draw, sample preparation and biochemical analyses have been previously published [22], [23].

#### HRQoL

Information on HRQoL was obtained using the self-administered SF-36 health survey version 2 (SF-36v2). The SF-36v2 is a generic measure of HRQoL and contains 36 questions covering eight functional domains, physical functioning, role-physical, body pain, general health, vitality, social functioning, role-emotional and mental health, with higher scores (range 0–100) reflecting better perceived health [24]. Individual domain scores were standardized to a mean of 50 and standard deviations of 10, with a score above 50 representing better than average health status and below 50 representing poorer than average health status. Two summary scores, PCS and MCS, were computed on the basis of the eight domain scores using regression weights derived from principal component factor analysis. SF-36v2 has been found to be reliable and valid in Singapore [25] and is available in four language versions for Singapore: English, Chinese, Malay and Tamil. We have previously found that the English and Chinese versions of the SF-36v2 are equivalent, while the Malay version is very likely to be equivalent with the English. We did not have enough data on the Tamil SF-36v2 to comment on its equivalence with the English SF-36v2. Hence, in this study, we used all language versions except the Tamil surveys.

#### Family functioning measure

It is a self-reported three-item scale that measures family relationship, with higher scores (range 0–100) reflecting better family functioning [26].

### Disease states

#### Diabetes Mellitus

Diabetes mellitus (diabetes) was defined as (1) fasting plasma glucose ≥7.0 mmol/l or (2) a known history of diabetes mellitus or (3) current use of anti-diabetic medications. Type 1 diabetes was excluded from the analysis. Individuals with diabetes were classified into three groups as – 1) those with undiagnosed diabetes (fulfilling criterion 1 but not 2 and 3), 2) those with diagnosed diabetes but not taking anti-diabetic medication, and 3) those with diagnosed diabetes and taking anti-diabetic medication.

#### Hypertension

Hypertension was defined as (1) systolic blood pressure ≥140 mm Hg or (2) diastolic blood pressure ≥90 mm Hg or (3) history of hypertension or (4) currently use of anti-hypertensive medication. Blood pressure readings were taken during the health examination with the participants seated and resting for at least five minutes. Two readings were obtained two minutes apart using an automatic blood pressure instrument (Dinamap Pro100V2; Criticon, Norderstedt, Germany). If the difference between these two readings was greater than 10 mmHg for systolic and 5 mmHg for diastolic, a third reading was recorded after 10 minutes. Subjects with a systolic blood pressure of 140 mmHg or more or a diastolic blood pressure of 90 mmHg or more at the health examination, with no history of hypertension or taking antihypertensive medication were considered as having undiagnosed hypertension. Similar to diabetes, individuals with hypertension were classified into three groups as – 1) those with undiagnosed hypertension, 2) those with diagnosed hypertension but not taking medication, and 3) those with diagnosed hypertension and taking medication.

#### Dyslipidemia

Dyslipidemia was defined as serum total cholesterol ≥6.2 mmol/l (1) or triglycerides ≥2.3 mmol/l (2) or low-density lipoprotein cholesterol ≥4.1 mmol/l (3) or high-density lipoprotein cholesterol <1.0 mmol/l (4), or history of dyslipidemia (5) or currently taking medication for dyslipidemia (6). As above, individuals with dyslipidemia were classified into three groups as – 1) those with undiagnosed dyslipidemia (fulfilling at least one of criteria 1-4 but not 5 and 6), 2) those with diagnosed dyslipidemia but not taking medication, and 3) those with diagnosed dyslipidemia and taking medication.

#### Comorbid conditions

Coronary heart disease, stroke, lung disease (including asthma), cancer, musculoskeletal illness, and mental illness were identified using self-report data from the health questionnaire. Peripheral arterial disease was defined as ankle-brachial index (ABI) of ≤0.9, or prior limb arterial revascularization, with ABI calculated as the ratio of the higher of the two systolic pressures (from posterior tibial and dorsalis pedis) at the ankle to the average of the right and left brachial artery pressures. Retinopathy was detected through retinal photography, graded using a modified scale based on Early Treatment Diabetic Retinopathy Study [27], [28]. Peripheral neuropathy was identified using monofilament sensory test and neurothesiometer, as a neurothesiometer reading of greater than 25V at any site (test sites - apex of the big toe and medial malleolus of both feet) [29], or monofilament sensory test of fewer than 4 of 5 points in either foot (test sites - distal great toe, third toe, and fifth toe and the first and fifth metatarsal heads of both feet) [30]. Chronic kidney disease was defined based on the presence of albuminuria and estimated glomerular filtration rate <60 mL/minute per 1.73 m^2^ (using the “modification of diet in renal disease” equation) [31].

### Statistical analysis

**Figure 1:**
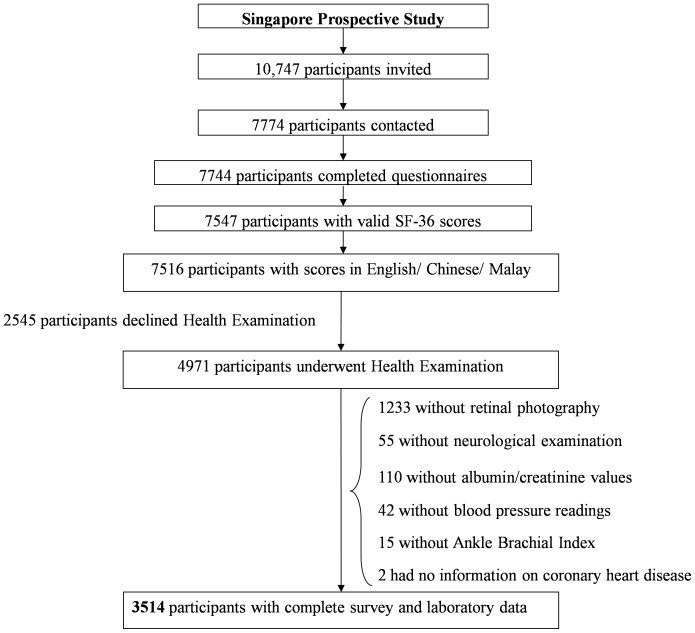
Inclusion of participants in the study.

Only those subjects with complete information to classify them for all of the following: diabetes status, dyslipidemia, hypertension, as well as key comorbid conditions (coronary heart disease, stroke, lung disease (including asthma), cancer, musculoskeletal illness, mental illness, peripheral arterial disease, peripheral neuropathy, chronic kidney disease and retinopathy), were included in the analysis. Subjects without responses to questions asking for history of diabetes, dyslipidemia, hypertension, coronary heart disease, stroke, lung disease (including asthma), cancer, musculoskeletal illness, and mental illness were excluded as were those without fasting plasma glucose, albumin and creatinine, retinal photos, neurological examination, ankle brachial index, and blood pressure readings. Among 7744 individuals who completed health questionnaires, 7547 had valid SF-36 scores. Of these 31 completed the survey in Tamil and were excluded. 2545 subjects declined health screening, while another 1457 had incomplete health screening data due to constraints as explained above. Thus 3514 individuals with complete interview and health examination data were included in the analysis (Figure 1). Table 1 compares characteristics of those included in the analysis and those excluded. Those excluded from analysis were slightly older, with higher proportion of self-reported diabetes and hypertension, but lower proportion of self-reported dyslipidemia.

**Table 1:** Characteristics of individuals included in and excluded from analysis.

Subject characteristics	Included (N = 3514)	Excluded (N = 4033)	P
Age, years	49 (11.28)	50 (13.61)	0.003
Male, N (%)	1680 (47.8)	1860 (46.1)	NS
Ethnicity, N (%)			<0.001
Chinese	2,074 (59.0)	2,795 (69.3)	
Malay	768 (21.9)	711 (17.6)	
Indian	672 (19.1)	527 (13.1)	
Currently married, N (%)	2,877 (81.9)	3,031 (75.2)	<0.001
Education, N (%)			<0.001
<7 years	844 (24.0)	1,296 (32.1)	
7–10 years	1,349 (38.4)	1,330 (33.0)	
>10 years	1,321(37.6)	1,407 (34.9)	
Currently working, N (%)	2,458 (69.9)	2,588 (64.2)	<0.001
Current smoker, N (%)	423 (12.0)	551 (13.7)	0.03
Current alcohol use, N (%)	789 (22.5)	841 (20.9)	NS
Self-reported Diabetes, N (%)	312 (8.9)	482 (12.0)	<0.001
Self-reported Hypertension, N (%)	729 (20.7)	965 (23.9)	0.001
Self-reported Dyslipidemia, N (%)	1060 (30.2)	1107 (27.4)	0.009
PCS	49.78 (9.54)	50.28 (10.18)	0.03
MCS	50.83 (9.53)	49.28 (10.32)	<0.001
Body Mass Index (N = 1474)	24.2 (4.33)	23.4 (4.20)	<0.001
Family Functioning Measure	61 (18.3)	57.3 (17.5)	<0.001

MCS – mental health component summary score; PCS – physical health component summary score.

All analysis in this paper was cross-sectional. Separate analyses were run to evaluate associations between disease awareness and HRQoL in diabetes, hypertension, and dyslipidemia. For each condition, individuals were categorized into 4categories;1) no disease – the reference category, 2) undiagnosed disease – for effect of the disease process itself, 3) diagnosed disease not taking medication –for effect of disease awareness, and 4) diagnosed disease with medication – for effect of disease awareness with treatment. One-way ANOVA was used to compare PCS and MCS scores across these categories. To adjust for the effects of other known determinants of HRQoL, multiple linear regression analyses were performed with PCS and MCS as outcome variables in separate models, and including disease awareness status, age, gender, ethnicity, marital status, education, occupation, smoking, alcohol intake, other comorbid conditions, body mass index and family functioning measure as covariates. Age was centred to the mean for all analyses.

ANCOVA was used to obtain adjusted levels of metabolic parameters (glycated hemoglobin, fasting plasma glucose, systolic and diastolic blood pressures, serum total cholesterol and serum triglycerides) across diagnosis categories, adjusted for age, gender, ethnicity, marital status, education, occupation, smoking, alcohol intake, other comorbid conditions, body mass index and family functioning measure. Stata 10 for Windows software (Stata Corporation, College Station, Texas) was used for all analyses. All statistical tests used were 2-sided, with a P<0.05 being considered as significant.

## Results

The mean (±SD) age of participants in the study was 49 (±11) years, with 47.8% being men (Table 2). 396 (11.3%) had diabetes, 1399 (39.8%) had hypertension, 1691 (48.1%) had dyslipidemia, and 1727 (49.1%) had at least one of following: coronary heart disease, stroke, peripheral arterial disease, peripheral neuropathy, retinopathy, nephropathy, lung disease, cancer, musculoskeletal illness, and mental illness.

**Table 2:** Characteristics of participants in the study.

Participant characteristics, N = 3514	Mean (SD)
Age, years	49 (11)
Male, N (%)	1680 (47.8%)
Ethnicity, N (%)	
Chinese	2074 (59%)
Malay	768 (21.9%)
Indian	672 (19.1%)
Marital status, N (%)	
Never married	380 (10.8%)
Currently married	2877 (81.9%)
Separated/Divorced/Widowed	257 (7.3%)
Education, N (%)	
<7 years	844 (24%)
7–10 years	1349 (38.4%)
>10 years	1321 (37.6%)
Currently employed, N (%)	2458 (69.9%)
Current smoker, N (%)	423 (12%)
Alcohol consumption, N (%)	789 (22.4%)
Diabetes mellitus, N (%)	396 (11.3%)
Hypertension, N (%)	1399 (39.8%)
Dyslipidemia, N (%)	1691 (48.1%)
Other conditions*, N (%)	1727 (49.1%)
Body mass index, kg/m2	24.2 (4.33)
Family Functioning Measure	61.01 (18.34)
PCS	49.78 (9.54)
MCS	50.83 (9.53)

MCS – mental health component summary score; PCS – physical health component summary score.

*Other conditions: coronary heart disease, stroke, peripheral arterial disease, peripheral neuropathy, retinopathy, nephropathy, lung disease, cancer, musculoskeletal illness, and mental illness.

### HRQoL and diagnosis status

#### Diabetes mellitus

Individuals with diagnosed diabetes, taking or not taking medication for diabetes, had significantly lower unadjusted PCS scores compared to those with no diabetes or undiagnosed diabetes. However, after adjustment for socio-demographics and other comorbities, only individuals with diagnosed diabetes not taking medication had significantly lower PCS scores (Table 3). Individuals with diagnosed diabetes taking medication were not significantly different from those with no diabetes. This suggests that while disease awareness is associated with reduced PCS, this effect is attenuated by medical therapy. While there was no difference in unadjusted MCS scores, individuals with undiagnosed diabetes had significantly higher MCS scores compared to those with no diabetes after adjustment.

**Table 3:** Associations between physical and mental component summary scores and disease awareness.

Condition	N	Unadjusted scores				Adjusted scores*			
		PCS		MCS		PCS		MCS	
		Mean (SD)	P	Mean (SD)	P	B (SE)	P	B (SE)	P
***Diabetes mellitus***									
No disease	3118	50.2 (9.1)	Ref	50.9 (9.5)	Ref	Ref		Ref	
Undiagnosed	84	49.5 (8.9)	NS	53.2 (9.1)	NS	0.6 (1.0)	NS	2.4 (1.0)	0.014
Diagnosed not taking medication	54	45.8 (12.2)	0.004	48.5 (9.4)	NS	−2.7 (1.2)	0.033	−1.9 (1.2)	NS
Diagnosed taking medication	258	45.5 (12.5)	<0.001	49.4 (10.3)	0.09	−0.8 (0.6)	NS	−0.8 (0.6)	NS
***Hypertension***									
No disease	2115	50.3 (9.0)	Ref	50.9 (9.5)	Ref	Ref		Ref	
Undiagnosed	670	50.1 (9.4)	NS	52.2 (9.3)	0.018	1.2 (0.4)	0.004	0.7 (0.4)	NS
Diagnosed not taking medication	116	50.3 (9.7)	NS	48.7 (8.9)	NS	0.9 (0.9)	NS	−2.6 (0.9)	0.002
Diagnosed taking medication	613	47.7 (11.2)	<0.001	49.6 (9.9)	0.015	0.5 (0.5)	NS	−1.4 (0.5)	0.004
***Dyslipidemia***									
No disease	1823	50.6 (8.9)	Ref	51 (9.4)	Ref	Ref		Ref	
Undiagnosed	631	50.1 (9.4)	NS	52.4 (9.5)	0.008	0.4 (0.4)	NS	0.8 (0.4)	0.045
Diagnosed not taking medication	649	48.95 (9.8)	0.001	49.9 (9.7)	0.077	−1.3 (0.4)	0.003	−0.9 (0.4)	0.032
Diagnosed taking medication	411	47.2 (11.6)	<0.001	49.3 (9.7)	0.009	−0.8 (0.5)	NS	−1.9 (0.5)	<0.001

MCS – mental health component summary score; PCS – physical health component summary score.

* – covariates in the model - age, gender, ethnicity, marital status, education, occupation, smoking, alcohol intake, other comorbid conditions, body mass index and family functioning measure.

#### Hypertension

As was observed for diabetes, PCS scores were also lower in those with diagnosed hypertension, although in this instance, the association was observed primarily in those taking medications for hypertension. As with diabetes, the association was no longer statistically significant after adjustment for socio-demographic factors and other comorbities (Table 3). Unadjusted MCS were lower in individuals with diagnosed hypertension taking medication and this remained significant in the multiple linear regression model. Adjusted MCS score was also significantly lower in individuals with diagnosed hypertension not taking medication, suggesting that disease awareness is associated with lower MCS in hypertension. Undiagnosed hypertension was associated with higher HRQoL. However, unlike in diabetes, the association was with PCS rather than MCS after adjustment for socio-demographic factors and other comorbities.

#### Dyslipidemia

Associations between dyslipidemia status and HRQoL had features in common with both diabetes and hypertension. Like diabetes, diagnosed dyslipidemia was associated with lower PCS scores, and these associations were attenuated after adjustment for socio-demographic and comorbid conditions, remaining statistically significant only for those who were not taking medication. However, the association between dyslipidemia status and MCS was more like that observed with hypertension, with lower MCS in those with known dyslipidemia and these associations were stronger after adjustment. These results suggest that disease awareness in dyslipidemia is associated with both lower PCS and MCS, with medical therapy attenuating only the reduction in PCS. Undiagnosed dyslipidemia was associated with increased MCS, both before and after adjustment.

Tables S1 to S3 show the relationships between diagnosis status and SF-36 sub-scales for all three conditions. Reduced PCS in those not taking medication appeared to be mainly driven by reduction in scores for bodily pain and physical functioning sub-scales in those with diabetes, and by bodily pain and general health sub-scales in those with dyslipidemia. All sub-scales mapping to MCS had significantly lower scores in those with dyslipidemia not taking medication, while none were significant in those with hypertension not taking medication. Higher MCS scores in those with undiagnosed diabetes and dyslipidemia were related to increased scores in the mental health and vitality sub-scales.

### Diagnosis status and extent of metabolic derangement

**Figure 2:**
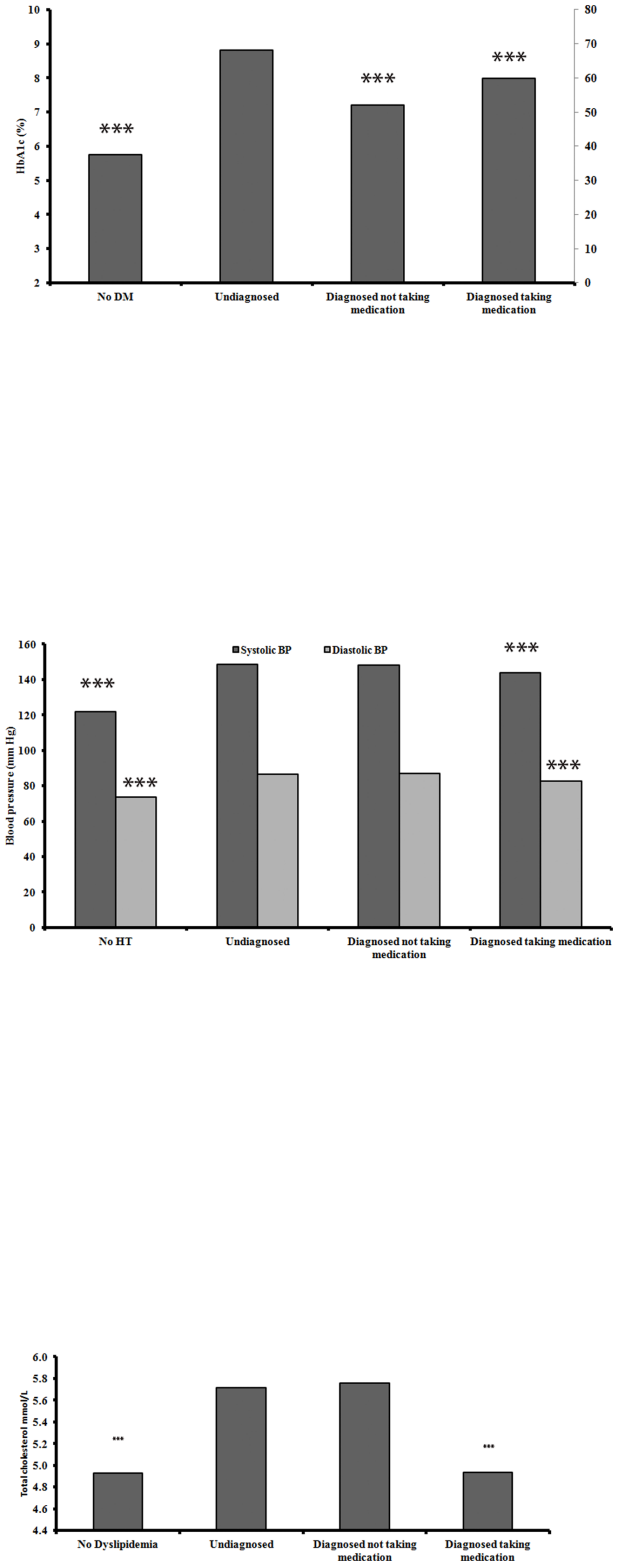
Metabolic derangement across disease awareness categories. a) Diabetes mellitus. b) Hypertension. c) Dyslipidemia. Reference category: Individuals with undiagnosed disease in each disease. *** - p<0.001.

We next examined whether disease severity, i.e. the extent of metabolic derangements, might influence the association between diagnosis status and HRQoL, to test the hypothesis that undiagnosed disease was not associated with HRQoL because of lower degree of metabolic derangement in those with undiagnosed disease (Figure 2). In comparisons across disease diagnosis categories, individuals with undiagnosed disease had similar or worse metabolic parameters compared to individuals with diagnosed disease, taking or not taking medication, in all three conditions. To further verify this, we divided subjects with undiagnosed disease into two sub-categories, based on the extent of metabolic derangements, and re-examined associations with PCS and MCS scores. As seen in Table S4, PCS and MCS scores in individuals from both categories of undiagnosed disease were either not different from those without disease, or higher compared to those without disease. Therefore, based on our data, it is unlikely that disease severity per se has an effect on HRQoL.

## Discussion

In this paper, we attempted to determine the relative contributions of disease awareness, treatment and comorbid conditions in the association between chronic disease and HRQoL.

In general, it is clear that individuals with undiagnosed disease did not experience any impairment of HRQoL. In fact, as we had suggested in a previous study [15], higher scores for HRQoL were observed in those with undiagnosed disease than those without disease. The specific domain (PCS or MCS) associated with undiagnosed disease differed somewhat between the three disorders and the strength of the association depended on whether or not covariates were adjusted for. These improved HRQoL scores could not be explained by the disease severity in terms of metabolic profiles, as profiles of individuals with undiagnosed disease were comparable or in some cases poorer than those with diagnosed disease. Individuals with worse metabolic profiles and undiagnosed disease had scores similar or higher than those without disease or mild metabolic impairments. Thus, the lack of association between undiagnosed disease and HRQoL might partly explain the high prevalence of undiagnosed diabetes, hypertension and dyslipidemia in this population [32] as individuals with “normal” or “enhanced” HRQoL may be less likely to present to their physicians or participate in screening programs so that the disease can be diagnosed.

The presence of diagnosed diabetes, hypertension or dyslipidemia was associated with lower PCS scores. For all three diseases, the associations were attenuated by adjustment for other covariates. This suggests that a proportion of the effects of these diseases on PCS are confounded by, or mediated through, other factors. We have previously reported that in individuals with diabetes, reduction in HRQoL is associated with the presence of complications rather than diabetes itself [16]. Our findings here seem to indicate that this is true of hypertension and dyslipidemia as well. These findings are similar to other studies which have looked at quality of life in patients with known disease [3]–[8]. Clearly, these associations with reduced PCS do not relate to the degree of metabolic perturbation observed in these patients since the metabolic profiles are better than those with undiagnosed disease. These associations are also unlikely to relate to medication use, since the associations are stronger in those not taking medications than in those taking medications, except in hypertension. It is, therefore, likely that these associations are due to either awareness of their disease among these individuals or some other factor we could not capture in our study.

Our findings with respect to medications in hypertension are in contrast to findings published earlier where individuals taking medication had worse HRQoL scores, both PCS and MCS, compared to those not receiving medication [17]. It is possible that these differences are due to differences in the type of antihypertensive agents prescribed and in use by subjects in that study and ours. Indeed, it has been shown that drugs belonging to the same class of agents with similar clinical profiles have vastly different effects on health related quality of life [33]. Also, individuals not taking medication included both those with diagnosed disease as well as those with undiagnosed disease, and comorbid conditions were not adjusted for in that study. Secondly, we report varying effects of medications in different diseases on MCS. The reasons for this are not clear, and need to be explored further. One possibility is the occurrence of adverse effects with lipid-lowering therapy which affect mental well-being. Indeed, newer studies on cholesterol lowering drugs report more adverse effects and effects on HRQoL [34]–[36], while older studies have reported no reductions in HRQoL due to therapy [37], [38]. Like with antihypertensive drugs, it is likely that these differences are also due to differing quality of life effects of the various drugs available and prescribed for dyslipidemia.

Our study has several strengths. We were able to characterise disease awareness and have complete data on self-reported medication status in a large population derived from the general community. We adjusted for ethnicity which may have cultural influence on the perception of diseases. We also adjusted for other comorbid conditions that might influence HRQoL.

There were several limitations worth mentioning. The cross-sectional nature of this study prevents examination of any causal relationship between disease awareness and medication in these conditions and HRQoL. Known disease status was obtained through self-report, which is subject to reporting and recall bias, and is therefore a limitation. Since this was a community survey, we were unable to include any examination of medical records to assess accuracy of such self-report, and this is a limitation of the study. Single time-point measurements were used to classify participants' disease status, unlike in the clinical setting, and therefore there is potential for erroneous classification of participants. However, other nationally representative surveys have also shown significant proportions of respondents with previously undiagnosed disease [32], [39], and therefore it appears that under-diagnosis of these conditions is a true rather than artefactual finding. The number of individuals with known diabetes and not taking medication was small. However, our findings in relation to HRQoL for all the three conditions are in agreement with a recent report in Thai population [15]. Therefore it is unlikely that the smaller numbers in this particular category affect the associations reported in this paper. It needs to be pointed out that though the differences in HRQoL scores after adjustment appear small, unadjusted difference in PCS scores ranged from 3–5 points, which is in the range deemed clinically significant [40]. Perceptions of illness and health are influenced by cultural contexts; therefore findings may not be fully generalizable to other populations. However, these findings still have direct relevance to populations with sizeable representation from Chinese, Indian or Malay ethnicities.

In summary, we have found that persons with diagnosed diabetes, hypertension or dyslipidemia have lower HRQoL, and this association is greatly influenced by presence of comorbid conditions. Importantly, treatment with medication, especially in diabetes and hypertension, was not associated with any adverse effect on HRQoL. This reinforces the importance of initiating treatment at the time of diagnosis, early in the natural history of these conditions, to prevent the development of comorbities. Equally importantly, individuals with undiagnosed disease have similar or better HRQoL compared to the non-diseased population. Thus a more robust implementation of the health screening strategy for cardiovascular disease and risk factors [41] is needed to detect and to treat these individuals early to prevent complications. At the same time, we need to consider strategies to limit the impact of disease awareness on HRQoL.

## Supporting Information

Table S1 **Associations between SF-36 sub-scales and diabetes status.**
(DOCX)

Table S2 **Associations between SF-36 sub-scales and hypertension status.**
(DOCX)

Table S3 **Associations between SF-36 sub-scales and dyslipidemia status.**
(DOCX)

Table S4 **Associations between physical and mental component summary scores and previously undiagnosed disease, categorized by severity of metabolic derangement.**
(DOCX)
