# Supplementary material for: Associations between Disease Awareness and Health-Related Quality of Life in a Multi-Ethnic Asian Population
Source: PLoS One. 2014 Nov 26;9(11):e113802. doi: 10.1371/journal.pone.0113802 (PMC4245227; doi:10.1371/journal.pone.0113802)
Supplement: Table S1 — Associations between SF-36 sub-scales and diabetes status. (DOCX) [file pone.0113802.s001.docx]

Table S1 – Associations between SF-36 sub-scales and diabetes status

| SF-36 sub-scale | Unadjusted scores | | | Adjusted scores* | | |
| --- | --- | --- | --- | --- | --- | --- |
|  | Mean | SD | p | B | SE | p |
| *Physical functioning* |  |  |  |  |  |  |
| No disease | 51.07 | 9.05 | Ref | Ref |  |  |
| Undiagnosed | 50.34 | 9.35 | 1 | 0.86 | 0.99 | 0.387 |
| Diagnosed not taking medication | 45.00 | 12.95 | <0.001 | -3.87 | 1.22 | 0.002 |
| Diagnosed taking medication | 45.11 | 12.11 | <0.001 | -1.57 | 0.62 | 0.012 |
| *Role physical* |  |  |  |  |  |  |
| No disease | 50.64 | 9.13 | Ref | Ref |  |  |
| Undiagnosed | 50.68 | 8.13 | 1 | 1.31 | 1.02 | 0.201 |
| Diagnosed not taking medication | 47.25 | 12.19 | 0.053 | -1.83 | 1.26 | 0.147 |
| Diagnosed taking medication | 45.62 | 12.16 | <0.001 | -1.60 | 0.64 | 0.013 |
| *Bodily pain* |  |  |  |  |  |  |
| No disease | 49.82 | 9.81 | Ref | Ref |  |  |
| Undiagnosed | 50.36 | 8.96 | 1 | 1.71 | 1.06 | 0.106 |
| Diagnosed not taking medication | 44.83 | 12.28 | 0.002 | -3.31 | 1.31 | 0.011 |
| Diagnosed taking medication | 45.78 | 11.73 | <0.001 | -0.14 | 0.67 | 0.829 |
| *General health* |  |  |  |  |  |  |
| No disease | 51.38 | 9.12 | Ref | Ref |  |  |
| Undiagnosed | 51.71 | 9.29 | 1 | 1.36 | 0.97 | 0.161 |
| Diagnosed not taking medication | 47.26 | 9.52 | 0.007 | -2.57 | 1.20 | 0.032 |
| Diagnosed taking medication | 46.77 | 10.66 | <0.001 | -2.16 | 0.61 | <0.001 |
| *Vitality* |  |  |  |  |  |  |
| No disease | 50.73 | 9.79 | Ref | Ref |  |  |
| Undiagnosed | 52.89 | 8.30 | 0.279 | 2.17 | 1.04 | 0.037 |
| Diagnosed not taking medication | 48.15 | 9.42 | 0.328 | -2.11 | 1.29 | 0.101 |
| Diagnosed taking medication | 49.03 | 10.36 | 0.044 | -0.62 | 0.66 | 0.347 |
| *Social functioning* |  |  |  |  |  |  |
| No disease | 50.52 | 9.38 | Ref | Ref |  |  |
| Undiagnosed | 50.49 | 9.14 | 1 | 0.75 | 1.04 | 0.473 |
| Diagnosed not taking medication | 48.23 | 10.27 | 0.485 | -1.44 | 1.29 | 0.263 |
| Diagnosed taking medication | 47.03 | 11.51 | <0.001 | -1.20 | 0.66 | 0.068 |
| *Role emotional* |  |  |  |  |  |  |
| No disease | 50.13 | 9.43 | Ref | Ref |  |  |
| Undiagnosed | 50.09 | 8.39 | 1 | 0.77 | 1.06 | 0.469 |
| Diagnosed not taking medication | 46.06 | 14.29 | 0.014 | -3.11 | 1.31 | 0.018 |
| Diagnosed taking medication | 47.43 | 11.84 | <0.001 | -0.26 | 0.67 | 0.695 |
| *Mental health* |  |  |  |  |  |  |
| No disease | 50.27 | 9.70 | Ref | Ref |  |  |
| Undiagnosed | 53.20 | 9.44 | 0.042 | 3.05 | 1.04 | 0.003 |
| Diagnosed not taking medication | 47.70 | 12.25 | 0.338 | -2.26 | 1.29 | 0.079 |
| Diagnosed taking medication | 49.76 | 10.64 | 1 | 0.45 | 0.66 | 0.494 |

* – covariates in the model - age, gender, ethnicity, marital status, education, occupation, smoking, alcohol intake, other comorbid conditions, body mass index and family functioning measure
