# Supplementary material for: Associations between Disease Awareness and Health-Related Quality of Life in a Multi-Ethnic Asian Population
Source: PLoS One. 2014 Nov 26;9(11):e113802. doi: 10.1371/journal.pone.0113802 (PMC4245227; doi:10.1371/journal.pone.0113802)
Supplement: Table S2 — Associations between SF-36 sub-scales and hypertension status. (DOCX) [file pone.0113802.s002.docx]

Table S2 – Associations between SF-36 sub-scales and hypertension status

| SF-36 sub-scale | Unadjusted scores | | | Adjusted scores* | | |
| --- | --- | --- | --- | --- | --- | --- |
|  | Mean | SD | p | B | SE | p |
| *Physical functioning* |  |  |  |  |  |  |
| No disease | 51.43 | 8.78 | Ref | Ref |  |  |
| Undiagnosed | 50.59 | 9.69 | 0.268 | 0.94 | 0.42 | 0.025 |
| Diagnosed not taking medication | 50.53 | 9.21 | 1 | 0.03 | 0.86 | 0.976 |
| Diagnosed taking medication | 47.30 | 11.11 | <0.001 | -0.19 | 0.49 | 0.689 |
| *Role physical* |  |  |  |  |  |  |
| No disease | 50.78 | 9.00 | Ref | Ref |  |  |
| Undiagnosed | 50.62 | 9.40 | 1 | 0.97 | 0.43 | 0.025 |
| Diagnosed not taking medication | 50.74 | 9.14 | 1 | 0.68 | 0.88 | 0.441 |
| Diagnosed taking medication | 47.73 | 10.94 | <0.001 | -0.39 | 0.50 | 0.436 |
| *Bodily pain* |  |  |  |  |  |  |
| No disease | 49.82 | 9.92 | Ref | Ref |  |  |
| Undiagnosed | 50.02 | 9.63 | 1 | 1.10 | 0.45 | 0.015 |
| Diagnosed not taking medication | 48.97 | 9.38 | 1 | -0.23 | 0.92 | 0.806 |
| Diagnosed taking medication | 47.68 | 10.87 | <0.001 | 0.62 | 0.52 | 0.238 |
| *General health* |  |  |  |  |  |  |
| No disease | 51.82 | 8.91 | Ref | Ref |  |  |
| Undiagnosed | 51.92 | 8.87 | 1 | 0.44 | 0.41 | 0.278 |
| Diagnosed not taking medication | 49.25 | 8.32 | 0.02 | -2.30 | 0.83 | 0.006 |
| Diagnosed taking medication | 47.42 | 10.51 | <0.001 | -2.67 | 0.48 | 0 |
| *Vitality* |  |  |  |  |  |  |
| No disease | 50.55 | 9.87 | Ref | Ref |  |  |
| Undiagnosed | 51.92 | 9.57 | 0.01 | 1.06 | 0.44 | 0.016 |
| Diagnosed not taking medication | 49.27 | 9.31 | 1 | -1.59 | 0.90 | 0.077 |
| Diagnosed taking medication | 49.67 | 9.83 | 0.3 | -0.47 | 0.51 | 0.363 |
| *Social functioning* |  |  |  |  |  |  |
| No disease | 50.31 | 9.54 | Ref | Ref |  |  |
| Undiagnosed | 51.46 | 8.83 | 0.04 | 1.48 | 0.44 | 0.001 |
| Diagnosed not taking medication | 49.28 | 9.96 | 1 | -0.77 | 0.90 | 0.393 |
| Diagnosed taking medication | 48.79 | 10.34 | 0.003 | -0.19 | 0.51 | 0.707 |
| *Role emotional* |  |  |  |  |  |  |
| No disease | 50.04 | 9.54 | Ref | Ref |  |  |
| Undiagnosed | 50.61 | 9.13 | 1 | 1.11 | 0.45 | 0.013 |
| Diagnosed not taking medication | 49.85 | 10.40 | 1 | 0.20 | 0.92 | 0.824 |
| Diagnosed taking medication | 48.46 | 10.68 | 0.002 | 0.07 | 0.52 | 0.889 |
| *Mental health* |  |  |  |  |  |  |
| No disease | 49.98 | 9.78 | Ref | Ref |  |  |
| Undiagnosed | 51.43 | 9.85 | 0.005 | 0.86 | 0.44 | 0.052 |
| Diagnosed not taking medication | 48.70 | 10.59 | 1 | -1.76 | 0.90 | 0.051 |
| Diagnosed taking medication | 50.29 | 9.72 | 1 | 0.07 | 0.51 | 0.886 |

* – covariates in the model - age, gender, ethnicity, marital status, education, occupation, smoking, alcohol intake, other comorbid conditions, body mass index and family functioning measure
