# Supplementary material for: Associations between Disease Awareness and Health-Related Quality of Life in a Multi-Ethnic Asian Population
Source: PLoS One. 2014 Nov 26;9(11):e113802. doi: 10.1371/journal.pone.0113802 (PMC4245227; doi:10.1371/journal.pone.0113802)
Supplement: Table S3 — Associations between SF-36 sub-scales and dyslipidemia status. (DOCX) [file pone.0113802.s003.docx]

Table S3 – Associations between SF-36 sub-scales and dyslipidemia status

| SF-36 sub-scale | Unadjusted scores | | | Adjusted scores* | | |
| --- | --- | --- | --- | --- | --- | --- |
|  | Mean | SD | p | B | SE | p |
| *Physical functioning* |  |  |  |  |  |  |
| No disease | 51.24 | 9.05 | Ref | Ref |  |  |
| Undiagnosed | 51.53 | 8.71 | 1 | 1.08 | 0.42 | 0.01 |
| Diagnosed not taking medication | 50.02 | 9.88 | 0.027 | -0.66 | 0.41 | 0.106 |
| Diagnosed taking medication | 46.56 | 11.12 | <0.001 | -1.35 | 0.53 | 0.011 |
| *Role physical* |  |  |  |  |  |  |
| No disease | 50.97 | 8.79 | Ref | Ref |  |  |
| Undiagnosed | 50.68 | 9.41 | 1 | 0.38 | 0.44 | 0.384 |
| Diagnosed not taking medication | 49.64 | 9.66 | 0.012 | -1.01 | 0.42 | 0.017 |
| Diagnosed taking medication | 47.08 | 11.60 | <0.001 | -1.68 | 0.55 | 0.002 |
| *Bodily pain* |  |  |  |  |  |  |
| No disease | 50.39 | 9.64 | Ref | Ref |  |  |
| Undiagnosed | 50.03 | 10.00 | 1 | 0.01 | 0.45 | 0.985 |
| Diagnosed not taking medication | 47.53 | 10.33 | <0.001 | -2.43 | 0.44 | 0 |
| Diagnosed taking medication | 47.50 | 10.78 | <0.001 | -0.85 | 0.57 | 0.136 |
| *General health* |  |  |  |  |  |  |
| No disease | 51.76 | 8.68 | Ref | Ref |  |  |
| Undiagnosed | 51.92 | 9.48 | 1 | 0.27 | 0.41 | 0.516 |
| Diagnosed not taking medication | 50.16 | 9.58 | 0.001 | -1.09 | 0.40 | 0.007 |
| Diagnosed taking medication | 47.41 | 10.55 | <0.001 | -2.77 | 0.52 | 0 |
| *Vitality* |  |  |  |  |  |  |
| No disease | 50.73 | 9.82 | Ref | Ref |  |  |
| Undiagnosed | 52.41 | 9.34 | 0.001 | 1.12 | 0.44 | 0.012 |
| Diagnosed not taking medication | 49.54 | 9.95 | 0.046 | -1.09 | 0.43 | 0.011 |
| Diagnosed taking medication | 49.07 | 9.81 | 0.011 | -1.81 | 0.56 | 0.001 |
| *Social functioning* |  |  |  |  |  |  |
| No disease | 50.67 | 9.45 | Ref | Ref |  |  |
| Undiagnosed | 50.83 | 9.21 | 1 | 0.49 | 0.44 | 0.274 |
| Diagnosed not taking medication | 49.51 | 9.53 | 0.049 | -0.92 | 0.43 | 0.034 |
| Diagnosed taking medication | 48.48 | 10.66 | <0.001 | -1.16 | 0.56 | 0.04 |
| *Role emotional* |  |  |  |  |  |  |
| No disease | 50.32 | 9.34 | Ref | Ref |  |  |
| Undiagnosed | 50.54 | 9.28 | 1 | 0.57 | 0.45 | 0.207 |
| Diagnosed not taking medication | 49.06 | 10.07 | 0.026 | -1.11 | 0.44 | 0.012 |
| Diagnosed taking medication | 48.09 | 11.15 | <0.001 | -1.08 | 0.57 | 0.06 |
| *Mental health* |  |  |  |  |  |  |
| No disease | 50.23 | 9.83 | Ref | Ref |  |  |
| Undiagnosed | 51.62 | 9.36 | 0.013 | 0.99 | 0.44 | 0.026 |
| Diagnosed not taking medication | 49.27 | 9.87 | 0.19 | -0.87 | 0.43 | 0.045 |
| Diagnosed taking medication | 49.88 | 10.20 | 1 | -0.89 | 0.56 | 0.114 |

* – covariates in the model - age, gender, ethnicity, marital status, education, occupation, smoking, alcohol intake, other comorbid conditions, body mass index and family functioning measure
