# Supplementary material for: Associations between Disease Awareness and Health-Related Quality of Life in a Multi-Ethnic Asian Population
Source: PLoS One. 2014 Nov 26;9(11):e113802. doi: 10.1371/journal.pone.0113802 (PMC4245227; doi:10.1371/journal.pone.0113802)
Supplement: Table S4 — Associations between physical and mental component summary scores and previously undiagnosed disease, categorized by severity of metabolic derangement. (DOCX) [file pone.0113802.s004.docx]

Table S4 – Associations between physical and mental component summary scores and previously undiagnosed disease, categorized by severity of metabolic derangement

|  |  | Unadjusted scores | | | | | | Adjusted scores* | | | | | |
| --- | --- | --- | --- | --- | --- | --- | --- | --- | --- | --- | --- | --- | --- |
|  | N | PCS | | | MCS | | | PCS | | | MCS | | |
| *Diabetes mellitus* |  | Mean | SD | P | Mean | SD | P | B | SE | P | B | SE | P |
| No disease | 3118 | 50.21 | 9.11 |  | 50.92 | 9.46 |  |  |  |  |  |  |  |
| Undiagnosed – Hba1c < 9% | 44 | 48.64 | 9.37 | 1 | 53.25 | 8.68 | 1 | -0.12 | 1.38 | 0.932 | 2.10 | 1.34 | 0.119 |
| Undiagnosed – Hba1c >= 9% | 40 | 50.47 | 8.45 | 1 | 53.18 | 9.56 | 1 | 1.52 | 1.44 | 0.292 | 2.69 | 1.41 | 0.056 |
| *Hypertension* |  |  |  |  |  |  |  |  |  |  |  |  |  |
| No disease | 2115 | 50.25 | 8.98 |  | 50.89 | 9.46 |  |  |  |  |  |  |  |
| Undiagnosed –  BP < 160/100 | 518 | 50.38 | 8.84 | 1 | 52.38 | 9.33 | 0.014 | 1.18 | 0.46 | 0.011 | 0.69 | 0.45 | 0.126 |
| Undiagnosed –  SBP ≥ 160 or DBP ≥ 100 mm Hg | 152 | 49.26 | 10.97 | 1 | 51.34 | 9.27 | 1 | 1.37 | 0.79 | 0.081 | 0.50 | 0.77 | 0.515 |
| *Dyslipidemia* |  |  |  |  |  |  |  |  |  |  |  |  |  |
| No disease | 1823 | 50.55 | 8.85 |  | 50.97 | 9.36 |  |  |  |  |  |  |  |
| Undiagnosed –  LDL-C < 4.9 and Tg < 5.6 mmol/L | 572 | 50.13 | 9.37 | 1 | 52.26 | 9.43 | 0.047 | 0.37 | 0.44 | 0.411 | 0.75 | 0.43 | 0.084 |
| Undiagnosed –  LDL-c ≥ 4.9 or TG ≥ 5.6 mmol/L | 59 | 50.15 | 10.00 | 1 | 53.55 | 9.99 | 0.397 | 0.67 | 1.20 | 0.573 | 1.65 | 1.17 | 0.158 |

* – covariates in the model - age, gender, ethnicity, marital status, education, occupation, smoking, alcohol intake, other comorbid conditions, body mass index and family functioning measure
